# Evaluating the two‐item measure of engagement at work for Japan Self‐Defense Forces: A cross‐sectional study

**DOI:** 10.1002/pcn5.70002

**Published:** 2024-08-21

**Authors:** Taku Saito, Masato Kitano, Fumiko Waki, Masanori Nagamine

**Affiliations:** ^1^ Division of Behavioral Science, National Defense Medical College Research Institute National Defense Medical College Saitama Japan

**Keywords:** Brief Job Stress Questionnaire, convergent validity assessment, internal consistency testing, job demands‐resources model, work engagement

## Abstract

**Aim:**

Work engagement is critical in both occupational and mental health contexts. However, no studies have compared the usefulness of the nine‐, three‐, and two‐item measures from the Utrecht Work Engagement Scale (UWES). Therefore, this study aimed to examine the internal consistency and convergent validity of the two‐item measure and compare its usefulness with the nine‐item and three‐item versions for assessing engagement at work among Japan Self‐Defense Forces (JSDF) personnel.

**Methods:**

This cross‐sectional study included 229,383 participants who underwent an annual mental health check between October 19 and December 17, 2021. To test the internal consistency of the scales, Cronbach's alpha was used. To test the convergent validity, Pearson's correlation coefficients were examined for each item corresponding to job resources, job satisfaction, stress reactions, and job demands assessed by the Brief Job Stress Questionnaire.

**Results:**

Most participants were men (89.8%). Cronbach's alpha coefficients for the nine‐, three‐, and two‐item scales were 0.95, 0.85, and 0.80, respectively. All three versions showed significant and positive correlations with each of the items corresponding to job resources and job satisfaction. The correlation coefficients of the two‐item scale were not inferior to those of the nine‐item and three‐item scales for job resources and job satisfaction items.

**Conclusion:**

Our results showed the internal consistency and convergent validity for the two‐item measure of engagement at work among JSDF personnel. The two‐item measure may be useful for briefly and efficiently assessing the actual state of workers' engagement at work.

## INTRODUCTION

*Work engagement* is a positive state of mind associated with work marked by three dimensions: *vigor*, *dedication*, and *absorption*.^1^, ^2^ According to the job demands–resources (JD–R) model,^3^ high job resources contribute to favorable results, such as superior job performance and organizational dedication, via work engagement.^4^, ^5^, ^6^ A recent meta‐analysis^7^ showed that work engagement was highly correlated with job satisfaction, in addition to job resources or personal resources. Therefore, work engagement plays a crucial role in both occupational and mental health.

The Utrecht Work Engagement Scale (UWES)^1^ is a commonly used scale for assessing work engagement around the world.^8^, ^9^, ^10^, ^11^, ^12^, ^13^, ^14^ The shortened version of the scale, the UWES‐9,^15^ consists of nine items, with three items each from the three subscales of work engagement. Additionally, the developers of the UWES demonstrated the reliability and validity of an ultrashort version (UWES‐3) consisting of three items, with one item from each of the three subscales (“At work, I feel like I am bursting with energy” [vigor]; “I am enthusiastic about my job” [dedication]; “I am immersed in my work” [absorption]).^16^

In Japan, the 2014 amended Industrial Safety and Health Act outlined a national policy to evaluate and improve working environments, and the Stress Check Program was launched in 2015.^17^ The program mandated that all workplaces with employees of 50 or more perform an annual examination of stress factors in workplaces, employees' mental and physical stress reactions, and the support received from supervisors and coworkers.^17^ The program manual recommended assessing these factors using the Brief Job Stress Questionnaire (BJSQ),^18^ an established Japanese stress scale to identify individuals experiencing high levels of stress. A new version of the BJSQ, the New BJSQ,^19^ was subsequently developed to assess working environments and outcomes more comprehensively by adding a scale of work engagement to the existing BJSQ scales, which included job stressors, buffering factors, psychological distress, and physical stress reactions.^19^ The reliability and validity of the New BJSQ have been demonstrated and fitted to the JD–R model.^3^, ^19^ During the process of developing the New BJSQ, considering the three work engagement subscales of the UWES, concerns were expressed about the negative effects of being too absorbed in one's work (workaholism).^20^ Therefore, the absorption item from the UWES was excluded, and work engagement was assessed by two items from the UWES^20^ in the New BJSQ: one each for the vigor and dedication items (“At work, I feel like I am bursting with energy” [vigor] and “I am proud of the work that I do” [dedication]).^19^ In the two items from the UWES, the vigor item is the same as the vigor item in the UWES‐3 (“At work, I feel like I am bursting with energy”). However, the dedication item in the two items from the UWES (“I am proud of the work that I do”) is different from the dedication item in the UWES‐3 (“I am enthusiastic about my job”). Numerous studies have been conducted so far using the BJSQ and the New BJSQ in the occupational health field in Japan^21^; however, no studies have examined the usefulness of the two items from the UWES to evaluate engagement at work.

The Ministry of Defense in Japan has conducted annual mental health checks for all Japan Self‐Defense Forces (JSDF) personnel since 2013 and has used the BJSQ to assess mental health and improve working environments since 2018. To create a more engaging working environment, the Ministry of Defense is seeking to test whether measuring work engagement can be used to assess JSDF personnel's level of engagement and improve their mental health. There is a wide range of occupations in the JSDF, such as uniformed personnel, clerical staff, and medical professionals in hospitals. However, the number of items from the UWES that is most useful for assessing work engagement among JSDF personnel remains unclear; to our knowledge, no previous studies have examined the usefulness of the two items from the UWES compared to the UWES‐9 and UWES‐3. Therefore, we considered that comparing the effectiveness of the nine‐, three‐, and two‐item measures in this population was academically important.

In this study, we aimed to examine the internal consistency and convergent validity of the two items from the UWES for assessing engagement at work among JSDF personnel and to compare the usefulness with UWES‐9 and UWES‐3 for efficiently assessing the actual state of their engagement at work. We hypothesized that the UWES‐9, UWES‐3, and two items from the UWES and job resources and job satisfaction assessed by the BJSQ would be positively correlated based on the JD‐R model.^3^ To achieve this, (1) correlation coefficients were calculated between the two items from the UWES and job resources, job satisfaction, stress reactions, and job demands assessed by the BJSQ to demonstrate convergent validity; (2) the internal consistency of the two items from the UWES was assessed; and (3) these results were compared with those of the UWES‐9 and UWES‐3.

## METHODS

### Study design and participants

This cross‐sectional study used the results of annual mental health checks of all JSDF personnel. Among the 229,383 JSDF personnel, those who responded to the BJSQ and UWES‐9 in the mental health checks conducted between October 19 and December 17, 2021, were included in this study.

This study was conducted in accordance with the Declaration of Helsinki and followed the COnsensus‐based Standards for the selection of health Measurement INstruments (COSMIN) guidelines.^22^, ^23^, ^24^ It was approved by the Institutional Review Board of the National Defense Medical College (approval number 4755).

### Measures

The Japanese version of the UWES‐9^11^, ^15^ and the BJSQ^18^ were used for the measurements.

#### The UWES

The UWES‐9 is a self‐administered questionnaire comprising nine items that measure work engagement. Each item is assessed using a 7‐point Likert scale, with responses ranging from 0 (*never*) to 6 (*always*). Higher scores indicate higher work engagement. The UWES‐3 and the two items from the UWES can be measured simultaneously by administering the UWES‐9 because the items of both measures are included in the UWES‐9. The scale was used for academic research purposes, following the guide of the original developer of the UWES and his Japanese official collaborator.

#### The BJSQ

The BJSQ^18^ is a 57‐item self‐administered Japanese questionnaire designed to assess job stressors, stress reactions, and buffering factors in accordance with the job stress model.^25^ Regarding job stressors, 17 items are rated on a 4‐point Likert scale ranging from 1 (*very much so*) to 4 (*not at all*). Five factors of this questionnaire correspond to job demands in the JD‐R model,^3^ including quantitative and qualitative job overload (three items each), physical demands (one item), interpersonal conflict (three items), and poor physical environment (one item); and four factors correspond to job resources in the JD‐R model,^3^ including job control (three items), skill utilization (one item), person–job fit (one item), and meaningfulness of work (one item). There are six stress reactions, measured through 29 items rated on a 4‐point Likert scale ranging from 1 (*almost never*) to 4 (*almost always*): lack of vigor (three items), anger/irritability (three items), fatigue (three items), anxiety (three items), depression (six items), and physical stress reaction (11 items). There are five buffering factors: support from supervisor, coworker, and family and friends (three items each), and satisfaction with job and family life (one item each). The nine items regarding three types of support are rated on a 4‐point Likert scale ranging from 1 (*extremely*) to 4 (*not at all*) and the two items regarding satisfaction with job and family life are rated on a 4‐point Likert scale ranging from 1 (*satisfied*) to 4 (*dissatisfied*). The rating score for each factor is calculated on a 5‐point Likert scale ranging from 1 (*low*) to 5 (*high*), according to Japan's Stress Check System Implementation Manual.^17^ To match the name of each factor with the direction of the evaluation score, we used the reciprocal number of the scores calculated based on the Stress Check Program Manual^17^ (arrived at by subtracting the scores calculated based on the manual from 5) for five job stressor factors (quantitative/qualitative job overload, physical demands, interpersonal conflict, and poor physical environment) and six stress reaction factors (lack of vigor, anger/irritability, fatigue, anxiety, depression, and physical stress reactions). This resulted in a rating score ranging from 1 to 5 for each factor. Higher scores indicated higher job stressors, stress reactions, and buffering factors.

### Statistical analysis

Cronbach's alpha coefficients were used to verify the internal consistency of these scales. Pearson's correlation coefficients were calculated between the scores of the UWES‐9, UWES‐3, and two items from the UWES and job resources (job control, skill utilization, person–job fit, meaningfulness of work, supervisor and coworker support), job satisfaction, satisfaction with family life, stress reactions (lack of vigor, anger/irritability, fatigue, anxiety, depression, and physical stress reactions), and job demands (quantitative/qualitative job overload, physical demands, interpersonal conflict, and poor physical environment) measured by the BJSQ to verify the convergent validity of these scales. All statistical analyses were conducted using R (Version 4.3.0) and R Studio.^26^ The significance threshold was set at *p* < 0.05.

## RESULTS

Among the 229,383 participants, the majority were men (89.8%). Table 1 shows the correlation coefficients of the UWES‐9, UWES‐3, and two items from the UWES. There were significantly high correlations between the measures. Cronbach's alpha coefficients for the UWES‐9, UWES‐3, and two items from the UWES were 0.95, 0.85, and 0.80, respectively.

**Table 1:** Pearson's correlation coefficients between measures.

	UWES‐9	UWES‐3	Two‐item measure of engagement at work
UWES‐9	1.00	0.96	0.94
UWES‐3		1.00	0.91
Two‐item measure of engagement at work			1.00

*Note*: All correlations were *p* < 0.001.

Abbreviation: UWES, Utrecht Work Engagement Scale.

Table 2 shows the correlations between the scores of the UWES‐9, UWES‐3, and two items from the UWES and the scores for job resources, job/life satisfaction, stress reactions, and job demands as assessed by the BJSQ. Job control, skill utilization, person–job fit, meaningfulness of work, supervisor support, and coworker support, which correspond to job resources, were significantly and positively correlated with the UWES‐9, UWES‐3, and two items from the UWES. For all job resources and job/life satisfaction items, the correlation coefficients of the two items from the UWES were not inferior to those of the UWES‐9 and UWES‐3.

**Table 2:** Correlations between the scores of the UWES‐9, UWES‐3, and two items from the UWES and BJSQ.

	*r* (95% CI)		
BJSQ	UWES‐9	UWES‐3	Two‐item measure of engagement at work
Job resources			
Job control	0.32 (0.32–0.33)	0.30 (0.29–0.30)	0.32 (0.31–0.33)
Skill utilization	0.27 (0.26–0.27)	0.27 (0.26–0.27)	0.30 (0.29–0.30)
Person–job fit	0.51 (0.50–0.51)	0.48 (0.48–0.49)	0.52 (0.51–0.53)
Meaningfulness of work	0.57 (0.57–0.58)	0.55 (0.55–0.56)	0.61 (0.60–0.61)
Supervisor support	0.41 (0.40–0.41)	0.39 (0.38–0.39)	0.41 (0.40–0.42)
Coworker support	0.40 (0.39–0.40)	0.38 (0.37–0.39)	0.41 (0.40–0.42)
Job/life satisfaction			
Job satisfaction	0.58 (0.58–0.59)	0.55 (0.55–0.56)	0.60 (0.60–0.61)
Satisfaction with family life	0.30 (0.29–0.30)	0.28 (0.27–0.29)	0.30 (0.29–0.31)
Stress reactions			
Lack of vigor^a^	−0.62 (−0.62–−0.61)	−0.59 (−0.59–−0.58)	−0.62 (−0.62–−0.61)
Anger/irritability^a^	−0.32 (−0.21–−0.31)	−0.28 (−0.28–−0.27)	−0.31 (−0.32–−0.31)
Fatigue^a^	−0.37 (−0.38–−0.36)	−0.32 (−0.33–−0.31)	−0.36 (−0.36–−0.35)
Anxiety^a^	−0.27 (−0.27–−0.26)	−0.23 (−0.23–−0.22)	−0.26 (−0.27–−0.26)
Depression^a^	−0.44 (−0.45–−0.43)	−0.40 (−0.41–−0.40)	−0.44 (−0.44–−0.43)
Physical stress reaction^a^	−0.33 (−0.33–−0.32)	−0.29 (−0.30–−0.29)	−0.32 (−0.32–−0.31)
Job demands			
Quantitative job overload^a^	−0.07 (−0.08–−0.07)	−0.02 (−0.03–−0.02)	−0.08 (−0.08–−0.07)
Qualitative job overload^a^	0.02 (0.01–0.02)	0.05 (0.04–0.06)	0.02 (0.01–0.02)
Physical demands^a^	0.04 (0.04–0.05)	0.05 (0.04–0.05)	0.05 (0.04–0.05)
Interpersonal conflict^a^	−0.36 (−0.36–−0.35)	−0.34 (−0.34–−0.33)	−0.37 (−0.38–−0.36)
Poor physical environment^a^	−0.23 (−0.24–−0.22)	−0.21 (−0.22–−0.21)	−0.23 (−0.24–−0.22)

*Note*: *r* denotes the Pearson correlation coefficient. All correlations were *p* < 0.001.

Abbreviations: BJSQ, Brief Job Stress Questionnaire; CI, confidence interval; UWES, Utrecht Work Engagement Scale.

^a^ Reciprocal score (arrived at by subtracting the scores calculated based on the manual from 5).

The UWES‐9, UWES‐3, and two items from the UWES showed significant negative correlations with each stress reaction item. None of the scales showed high correlation coefficients with quantitative/qualitative job overload or physical demands, which correspond to job demands. There were significant negative correlations between interpersonal conflict and poor physical environment.

## DISCUSSION

The purpose of this study was to examine the internal consistency and convergent validity of the two items from the UWES and compare the usefulness with UWES‐9 and UWES‐3 for assessing engagement at work among JSDF personnel. The results of this study showed internal consistency and convergent validity for the two‐item measure of engagement at work among JSDF personnel. Although the two‐item measure of engagement at work consists of fewer items than the UWES‐9 and UWES‐3, its correlations with job resources and job satisfaction were comparable to those of the UWES‐9 and UWES‐3, suggesting that the two‐item measure may be useful for assessing engagement at work among JSDF personnel.

One major difference between the two‐item measure of engagement at work, UWES‐9, and UWES‐3 is that the two‐item measure does not include the Absorption subscale of work engagement. Therefore, it is unclear whether this two‐item measure can comprehensively measure work engagement. There is a downside to not being able to assess the domain of absorption. Work engagement was originally proposed as the opposite of burnout, with vigor identified as the opposite of exhaustion and dedication as the opposite of cynicism in burnout.^1^ However, absorption is not a direct counterpart concept to reduced efficacy in burnout; reduced efficacy in burnout and absorption in work engagement were both added later in the process of creating each psychological measure.^1^ Therefore, absorption may be less significant than vigor and dedication as a core concept of work engagement. Bakker et al.^27^ pointed out that absorption in work engagement may lead to unhealthy behaviors, as people might become so engaged in their work that they overlook the need to rest or maintain personal relationships. They argued that further research is needed to examine whether absorption is a core aspect of work engagement.^27^ In a cross‐sectional study of 1917 individuals aged 45 years and older in Australia, absorption in work engagement was negatively associated with proactive health behaviors, suggesting negative aspects of absorption.^28^ Furthermore, in the Japanese version of the UWES, absorption questions (e.g., “I feel happy when I am working intensely” or “I am immersed in my work”) may not provide a positive impression of work in the Japanese context and may instead be associated with workaholism. Thus, we posit that the two‐item measure—which does not include the Absorption subscale—may be useful for assessing engagement at work among JSDF personnel.

Cultural differences can influence work engagement. A cross‐sectional study comparing UWES‐9 scores among employees from 16 countries found that Japanese employees demonstrated significantly lower scores than employees in all other countries.^29^ The results of the study could indicate the tendency of Japanese individuals to restrain themselves from emphasizing positive aspects because it is considered important in Japanese society to maintain social harmony without asserting oneself.^29^ In an international cross‐sectional study of 10 countries mostly located in the West, the UWES‐9 was best fitted with a three‐factor structure,^15^ whereas in a study of Japanese participants, a one‐factor structure was best fitted.^11^ Therefore, when using work engagement scales, the influences of and differences in a country's culture must be considered. Although the two‐item measure of engagement at work was useful among JSDF personnel in our study, further research is needed to determine whether this is a unique trend for JSDF personnel with a special mission (e.g., national defense or emergency response) or whether it can be generalized to all Japanese workers.

In this study, the UWES‐9, UWES‐3, and two‐item measure of engagement at work showed significant positive correlations with each job resource factor, suggesting that they all fit the JD–R model.^3^ The results showed significant negative correlations with all factors related to stress reactions, supporting a previous study,^16^ which showed a negative correlation with psychological distress. Conversely, although weak (very low correlation coefficients) correlations were observed with some factors (quantitative/qualitative job overload and physical demands) in job demands, significant negative correlations (low correlation coefficients) were observed with other factors (interpersonal conflict and poor physical environment). In accordance with the JD–R model, the direction of the association with work engagement differs depending on the nature of job demands,^30^ and the results of this study are consistent with this—that is, interpersonal conflict and poor physical environment were negatively associated (although the correlation coefficients were low) with work engagement because they could hinder job demands. However, quantitative/qualitative job overload and physical demands were weakly correlated (the correlation coefficients were very low) with work engagement in this study because they could be challenging job demands. Furthermore, these results might reflect the organizational culture of the JSDF, which, as an organization that responds to emergencies, requires training to be prepared for harsh environments.

This study has some limitations. First, this study only examined internal consistency and convergent validity. Therefore, a complete assessment to validate the psychological scale following COSMIN guidelines is lacking.^22^, ^23^, ^24^ In addition, we calculated Cronbach's alpha in a two‐item measure. Although numerous studies reported Cronbach's alpha for the two‐item scale, there is concern about underestimating the true reliability.^31^ As this was a cross‐sectional study, it was not possible to show the test–retest reliability. We are currently planning a longitudinal study using the UWES that may validate its fit to the JD–R model. Second, the participants of this study were JSDF personnel and are not representative of all Japanese workers. The results should be interpreted carefully because the proportion of women in this study was only approximately 10%. However, Schaufeli et al.^15^ reported that gender differences in work engagement are not meaningful. In addition to uniformed JSDF personnel, a wide variety of occupations was represented, including JSDF clerical personnel or medical personnel working in hospitals. The large dataset of approximately 230,000 JSDF participants is a strength of this study; however, future comparative studies using the two‐item measure of engagement at work for other workers in Japan would be useful to extend the generalizability of our findings.

Despite the above limitations, this study showed internal consistency and convergent validity for the two‐item measure for assessing engagement at work among JSDF personnel. In particular, we found that the two‐item measure may be useful for briefly and efficiently assessing the actual state of JSDF personnel's engagement at work.

## AUTHOR CONTRIBUTIONS

All authors conceived the ideas; Taku Saito, Masato Kitano and Fumiko Waki collected the data; Taku Saito analyzed the data; and Masanori Nagamine supervised the study. All authors participated in interpretation of the results, writing the manuscript, and approved the final version.

## CONFLICT OF INTEREST STATEMENT

The authors declare no conflict of interest.

## ETHICS APPROVAL STATEMENT

This study was approved by the Institutional Review Board of the National Defense Medical College (approval number 4755).

## PATIENT CONSENT STATEMENT

This study was conducted on an opt‐out basis, and written informed consent was not required due to the retrospective observational study design using existing information.

## CLINICAL TRIAL REGISTRATION

N/A.

## Supporting information

Supporting information.

## Data Availability

The data of this study are available from the corresponding author, T.S., on reasonable request.
