# Supplementary material for: Evaluating the two‐item measure of engagement at work for Japan Self‐Defense Forces: A cross‐sectional study
Source: PCN Rep. 2024 Aug 21;3(3):e70002. doi: 10.1002/pcn5.70002 (PMC11339131; doi:10.1002/pcn5.70002)
Supplement: Supplementary file 1 — Supporting information. [file PCN5-3-e70002-s001.docx]

Supplementary Table 1. The bias of this study based on the COSMIN Risk of Bias checklist (1-3)

| Risk of bias | Assessment |
| --- | --- |
| 1. PROM development | Not applicable |
| 1. Content validity | Not applicable |
| 1. Structural validity | Not applicable |
| 1. Internal consistency | Very good |
| 1. Cross‐cultural validity/Measurement invariance | Not applicable |
| 1. Reliability | Not applicable |
| 1. Measurement error | Not applicable |
| 1. Criterion validity | Not applicable |
| 1. Hypotheses testing for construct validity |  |
| 9a. convergent validity | Very good |
| 9b. discriminative validity | Not applicable |
| 1. Responsiveness | Not applicable |

Abbreviation: COSMIN, COnsensus-based Standards for the selection of health Measurement Instruments; PROM, patient-reported outcome measure.**References**

1. Mokkink LB, De Vet HCW, Prinsen CAC, Patrick DL, Alonso J, Bouter LM, et al. COSMIN risk of bias checklist for systematic reviews of patient-reported outcome measures. Qual Life Res. 2018;27:1171-1179.

2. Prinsen CAC, Mokkink LB, Bouter LM, Alonso J, Patrick DL, De Vet HCW, et al. COSMIN guideline for systematic reviews of patient-reported outcome measures. Qual Life Res. 2018;27:1147-1157.

3. Terwee CB, Prinsen CAC, Chiarotto A, Westerman MJ, Patrick DL, Alonso J, et al. COSMIN methodology for evaluating the content validity of patient-reported outcome measures: a Delphi study. Qual Life Res. 2018;27:1159-1170.
